# Dark Septate Endophytes Improve the Growth and the Tolerance of *Medicago sativa* and *Ammopiptanthus mongolicus* Under Cadmium Stress

**DOI:** 10.3389/fmicb.2019.03061

**Published:** 2020-01-28

**Authors:** Lifeng Hou, Jie Yu, Lili Zhao, Xueli He

**Affiliations:** College of Life Sciences, Hebei University, Baoding, China

**Keywords:** dark septate endophytes, plant growth, cadmium tolerance, *Medicago sativa*, *Ammopiptanthus mongolicus*, symbiosis

## Abstract

Although the ecological function of dark septate endophytes (DSEs) is well studied, little is known about the responses of the host plant to DSEs obtained from other plants, especially under conditions of heavy metal stress. This study aimed to investigate how DSEs from a heavy-metal habitat affect non-host plants in cadmium (Cd) stress soils, which then provides a basis for the application of DSEs in the cultivation of different plant and soil remediation strategies for polluted ecosystems. We isolated and identified two species of DSE (*Acrocalymma vagum* and *Scytalidium lignicola*) inhabiting the roots of *Ilex chinensis* (host plant) which are grown in metal-polluted habitats. Then, the Cd stress tolerance of the DSEs was tested using a pure culture of which the Cd concentration has been adjusted. Subsequently, we examined the performance of non-host plants (*Medicago sativa* and *Ammopiptanthus mongolicus*) which were inoculated with DSEs under Cd stress in a growth chamber. The results indicated that the two DSEs could grow under Cd stress *in vitro*, even when not exhibiting high levels of tolerance to Cd. The superoxide dismutase (SOD), malondialdehyde (MDA), glutathione (GSH), soluble protein, and melanin of the DSE fungi reached maximal levels at concentrations of 30–60 mg Cd/L, indicating the important preventive strategies adopted by the DSE fungi in environments contaminated by Cd. Despite a decreased biomass of DSE hyphae with enhanced Cd concentrations, the accumulation of Cd in the DSE hyphae tended to show an increasing trend. Both DSEs were effective colonizers of the non-host plants. *A. vagum* and *S. lignicola* inoculation significantly promoted the biomass and the root architecture of the two non-host plants under Cd stress. *A. vagum* inoculation increased the total nitrogen (TN) of *A. mongolicus*, whereas inoculation with *S. lignicola* significantly increased the organic carbon (OC) of *M. sativa*. In particular, the DSE inoculation significantly improved the accumulation of Cd in plant tissues under Cd stress, demonstrating a potential application in the bio-remediation of heavy-metal-pollution areas. Our findings suggest that the DSE inoculation improved the root growth and nutrient absorption of non-host plants, altered the soil Cd concentration, and facilitated plant growth and survival under Cd stress. These results contribute to a better understanding of DSE–plant interactions in habitats contaminated by heavy metals.

## Introduction

Heavy metal pollution adversely affects the growth of plants and the soil ecosystem (Gallego et al., 2012). The plants respond both directly and indirectly to the changes in the soil’s environment. Nevertheless, indirect plant responses to environmental stress caused by fungal symbionts have been receiving increased levels of research attention over the recent years. Previous studies have reported that the association between the symbiotic fungi and the host plants may influence the response of plants to heavy metal stress and improve the resistance of plants to various environmental stresses (Li et al., 2011; He et al., 2017). Microbe-assisted phytoremediation has been considered as the most promising technology for the remediation of contaminated soils (Ashraf et al., 2017; Deng and Cao, 2017). Hence, it is important to identify more beneficial fungi that can be used in the cultivation of various plants and in soil remediation strategies for polluted ecosystems.

Dark septate endophytes (DSEs) belong to a diverse group of ascomycetes, which can form melanized septate hyphae and microsclerotia in the root tissue of plants (Jumpponen and Trappe, 1998; Sieber and Grünig, 2013). These assemblages have been found in almost all natural ecosystems, particularly in stressful environments (for example, polluted habitats and alpine, saline, and arid ecosystems). In some cases, several DSEs have been reported to exhibit tolerance to stressors under *in vitro* culture conditions (Likar and Regvar, 2013; Zhan et al., 2015b; Berthelot et al., 2016). Fungal melanin, as a complex biological macromolecular compound composed of phenolic and aromatic compounds, is believed to support the structural rigidity of the cell wall and may enhance the tolerance of fungi to various stressors, such as heavy metals, drought, and oxidative conditions (Butler and Day, 1998; Eisenman and Casadevall, 2012; Berthelot et al., 2017). Zhu et al. (2018) previously showed that antioxidant enzymes, such as superoxide dismutase (SOD), peroxidase, and catalase, can be synthesized by the DSE fungi to prevent oxidative damage under heavy metal stress. Moreover, the DSEs improve plant growth by promoting the plant uptake of C, N, and P (Mandyam and Jumpponen, 2005; Newsham, 2011) and by protecting the plants against biotic and abiotic stresses (such as pathogens, elevated carbon dioxide levels, heavy metals, and limited water supply) (Alberton et al., 2010; Hamilton et al., 2012; Berthelot et al., 2016; Li et al., 2019).

A rich variety of DSEs is associated with the roots of many species of host plants growing in habitats polluted by metals (Deram et al., 2011; Newsham, 2011). Previous studies have shown that the DSEs are frequent colonists of plant roots in heavy-metal-contaminated environments (Likar and Regvar, 2009; Zhang et al., 2013). In addition, the DSEs impede the transport of heavy metal ions from the roots to the shoots of maize (Li et al., 2011) and chelate cadmium (Cd) in the roots of the plants and also reduce Cd content in the tissues of shoots (Hui et al., 2015; Ban et al., 2017). Thus, the DSE fungi might play important roles in the remediation of contaminated soils and heavy-metal ecosystems. Interestingly, heavy metal contamination reduces the mycorrhizal colonization of *Salix caprea* but does not affect the DSE colonization (Likar and Regvar, 2009). Similarly, several studies have reported that the colonization of arbuscular mycorrhizal (AM) fungi is reduced under conditions of severe metal contamination, but no effects on the DSE have been observed (Ruotsalainen et al., 2007; Deram et al., 2011). Despite numerous indications relating to the worldwide distribution and diversity of DSEs in the literature, the ecological functions of the DSEs and the DSE–plant interactions in heavy-metal-contaminated habitats still need to be elucidated.

The distribution and the abundance of the DSEs in metal-polluted ecosystems have been widely investigated (Likar and Regvar, 2009; Deram et al., 2011; Berthelot et al., 2016). Recently, the influence of heavy metal stress on the growth of forage grass and ornamental plants has attracted increasing attention (Yang et al., 2015; Cui et al., 2018). Choosing and applying beneficial DSE fungi that are able to confer positive effects to plants in heavy-metal-contaminated soils appear to be necessary. The DSEs have been demonstrated to exhibit positive effects on non-host plants in an arid desert environment (Li et al., 2018). Nevertheless, little is known about the effects of DSEs obtained from other plants on non-host plant responses to heavy metal stress.

This study aimed to investigate how the DSEs from a host plant (*Ilex chinensis*) grown in habitats polluted by heavy metals affect the growth of non-host plants (*Medicago sativa* and *Ammopiptanthus mongolicus*) under Cd stress. First, two DSE strains were isolated from the roots of *I. chinensis* grown in metal-polluted habitats. Second, the isolated DSE strains were exposed to different concentrations of Cd in pure cultures to test their tolerance to Cd stress. Third, we investigated the effects of the DSE inoculation on the growth of non-host plants in an inoculation experiment using the DSE strains under conditions of Cd stress. Further, we addressed the following questions: (1) Do DSE strains from heavy-metal-contaminated environments exhibit high tolerance to Cd stress *in vitro*? (2) Could inoculation with the DSE fungi facilitate the growth of non-host plants under Cd stress? If so, (3) could Cd stress influence the association between plants and DSE fungi?

## Materials and Methods

### Fungi Materials

Root samples of *I. chinensis* Sims were collected from the Fengfeng mining area (36°28′ N, 114°10′ E) with metal pollution in Hebei Province, China. The root samples of *I. chinensis* were randomly selected, and the surface was disinfected by sequential washes in 75% ethanol (4 min) and 10% NaClO (2 min). These segments were rinsed several times in sterilized water and then dried on sterile filter paper. Finally, the root segments were transferred to a potato dextrose agar (PDA) culture medium with antibiotic supplements (ampicillin and streptomycin sulfate) in Petri dishes. The sterilized root segments were incubated in the dark at 27°C and were observed daily. To make sure that the isolates are true endophytes, 200 μL of the distilled water left in the final step was coated on the PDA medium as a contrast. The mycelium growing from the cut ends of the root segments were transferred to new PDA plates and kept in the dark at 27°C. The colony morphology on PDA and the microscopic morphological characteristics of the isolates were observed (Li et al., 2018).

These fungi were identified based on the phylogenetic analysis of nrDNA Internal Transcribed Spacer (ITS) sequences according to the method described by Xie et al. (2017). Briefly, DNA was extracted from the fresh hyphae (approximately 50 mg) of each colony using a genomic DNA extraction kit (Solarbio, China). The universal primers were ITS4 (5′-TCCTCCGCTTATTGATATGC-3′) and ITS5 (5′-GGAAGTAAAAGTCGTAACAAGG-3′). The reaction system (20 μL) included 3.5 μL of DNA template, 0.5 μL of each 10 μM primers, 10 μL of 2 × Es Taq Master Mix, and 5.5 μL of ddH_2_O. PCR was performed in a Life ECO^TM^ (BIOER, China) using the following program: initial denaturation at 94°C for 5 min, then 35 cycles of denaturation at 94°C for 1 min, primer annealing at 55°C for 1 min, extension at 72°C for 1 min, and then a final extension at 72°C for 10 min. The PCR products were purified and sequenced. The sequences were deposited in GenBank under the accession numbers MH410647.1 (DSE1) and MH410648.1 (DSE2). Clustal X (v.1.81) was used to perform the sequence alignment, and the maximum likelihood tree was drawn with MEGA 6 (Tamura et al., 2013). The two isolates (DSE1 and DSE2) were preserved in the culture collection of the Laboratory of Plant Ecology, Hebei University, China. All isolates were cultured on the PDA medium for 2 weeks under dark conditions at 27°C for subsequent experiments.

### Plant Materials

*Medicago sativa*, which belongs to Leguminosae, is an important perennial forage grass grown widely all over the world. *A. mongolicus*, a legume evergreen broad-leaf ornamental shrub characterized by rapid growth and high drought resistance, has been widely used in vegetative restoration and landscape greening in northwest China. In this study, *M. sativa* and *A. mongolicus* were chosen as the inoculated plants (Li et al., 2015). The seeds of *M. sativa* were supplied by Hebei Agriculture University, China. The ripe seeds of *A. mongolicus* were collected from natural populations in northwest China. All of the seeds were stored at 4°C until pre-germination.

### DSE Tolerance to Cd Stress *in vitro*

The capacity of the DSE isolates to grow under Cd stress was tested in a preliminary experiment. The experiment was performed under sterile conditions using a modified Melin–Norkrans (MMN) medium (pH 5.5). Twenty grams of agar was added to solidify the MMN medium. An appropriate amount of CdCl_2_ ⋅ 2.5H_2_O stock solution was added to the MMN to obtain a series of media that contain varying concentrations of Cd. Fungal disks (9 mm in diameter) were then cut from the edge of the DSE colonies that have been cultured for 14 days. Each disk was placed as an inoculum on different culture plates and in different flasks, with five replicates per treatment (one disk per replicate). On the culture plates, the concentrations of Cd were set to 0, 10, 30, and 50 mg/L. The fungi were cultured at 27°C for 14 days. The morphological and microscopic features of the colonies on MMN were observed. In the liquid media, the concentrations of Cd were 0, 10, 20, 30, 40, 50, and 60 mg/L. The fungal disks were inoculated in a 250-ml conical flask containing 150 mL of liquid medium and cultured for 14 days at 27°C, with rotation at 160 r/min. After culturing in liquid media, the fungal mycelia were first collected by a suction filter. The fresh mycelia were then randomly divided into two portions. One portion was used to determine the levels of SOD, malondialdehyde (MDA), glutathione (GSH), soluble protein, and melanin. The other portion was weighed and then dried at 80°C to a constant weight so that the water content could be determined. The sum of the dry weights of the two portions was considered to represent the total biomass of the DSE.

### Determination of Soluble Protein, Glutathione, Melanin, and Cd Content

Soluble protein concentration was measured using the method of Coomassie Brilliant Blue G-250 reagent as described by Li and Jiao (1980). Briefly, the fresh hyphae (0.2 g) were ground into homogenate with the aid of quartz sand in 5 mL of 50 mM ice-cold phosphate buffer (pH 7.0) at 4°C in a pre-cooled mortar. The homogenate was centrifuged at 4000 × *g* for 10 min at 4°C, and then the supernatant was collected. The absorbance of the reaction mixture was measured at 595 nm via a spectrophotometer (752N model, Shanghai INESA Instrument Analytical Instruments Co., Ltd., China). Bovine serum albumin was used as a standard.

The GSH concentration was determined by the method of dithiobis-nitrobenzoic acid as described by Anderson (1985). Briefly, the fresh hyphae (0.2 g) were homogenized at 4°C in 5% (*w*/*v*) sulfosalicylic acid. After centrifugation for 10 min at 10,000 × *g*, the GSH concentration was measured, using a spectrophotometer, by the absorbance of the supernatant at 421 nm according to a standard curve.

Hyphal melanin was extracted using the method of Ellis and Griffiths (1974). The main process is as follows: First, the fresh hyphae (0.2 g) were heated in 1 M NaOH at 100°C for 4 h. Second, the cooled extract was filtered and acidified with 7 M HCl until precipitation at pH 2. Third, after centrifugation for 15 min at 10,000 × *g*, the precipitate was recovered and washed with distilled water. The coagulated melanin was then dissolved in 1 M NaOH, and the yield of melanin was estimated. The melanin concentration was measured by a standard curve plotted from the results of photometry at 459 nm.

The mass fractions of Cd^2+^ in the DSE hyphae were determined. The dried hyphae (approximately 0.05 g) were digested in a digestion solution of HNO_3_/HClO_4_ (3:1) (*v*/*v*). The digestion solutions were diluted with deionized water to 25 mL. The mass fractions of Cd^2+^ in the DSE hyphae were determined using a flame atomic absorption spectrometer (TAS-990, Beijing Puxi Instrument Factory, Beijing, China).

### Determination of Superoxide Dismutase Activity and Malondialdehyde Concentration

The activity of SOD was assayed by the photochemical method described by Elavarthi and Martin (2010). The fresh hyphae (0.2 g) were ground into a homogenate with 5 mL of 50 mM phosphate buffer (including 0.2 mM EDTA and 2% polyvinylpyrrolidone) (pH 7.8) at 4°C in a pre-cooled mortar. The homogenate was centrifuged at 15,000 × *g* and 4°C for 30 min, and then the supernatant was collected for the analysis of enzyme activity. One unit (U) of SOD activity was defined as the amount of enzyme that caused 50% inhibition of photochemical reduction of NBT at a wavelength of 560 nm.

The MDA concentration was assayed by the thiobarbituric acid (TBA) method according to Peever and Higgins (1989). Briefly, the fresh hyphae (0.2 g) were homogenized in 10% trichloroacetic acid (5 mL) and centrifuged at 12,000 × *g* for 10 min. The mixture containing the supernatant (2 mL) and 0.5% TBA (2 mL) was placed in a boiling water bath. After 15 min, the mixture was rapidly cooled and then centrifuged at 12,000 × *g* for 10 min. The absorbance of the supernatant was determined at 450, 532, and 600 nm using a spectrometer. The following formula was used to calculate the MDA concentration:

C(μmol/L)=6.45(OD-532OD)600-0.56OD450

### Plant Growth Promotion Experiment

The experiment was conducted in a growth chamber using a completely randomized design in a 3 × 3 factorial arrangement with the various DSE inoculation treatments (non-inoculated control, *Acrocalymma vagum*, and *Scytalidium lignicola*) and concentrations of Cd stress treatments (0, 5, and 10 mg Cd/kg soil) as the variables for each plant species (*M. sativa* and *A. mongolicus*). Each treatment had five repetitions; thus, a total of 90 experimental pots were set up.

The seeds from each species of plant were surface-sterilized with 70% ethanol for 3 min and with 2.5% sodium hypochlorite for 10 min under agitation and then rinsed three times with sterile water. The sterilized seeds were then placed on a water agar medium (containing 10 g/L agar) for germination at 27°C. Following germination, the seedlings were planted in sterile pots (5.5 cm in diameter at the base, 8.5 cm in diameter at the top, and 11.5 cm in height). For *M. sativa*, two seedlings were transferred to each pot. For *A. mongolicus*, one seedling was planted in each pot. Approximately 400 g of culture substrata (200 g of soil mixed with 200 g of river sand) was placed in each pot. The culture substrata was autoclaved for 120 min at 121°C and contained 13.76 mg/g of organic matter, 130 mg/kg of ammonium nitrogen, 5.58 mg/kg of available phosphorus, 570 mg/kg of total nitrogen (TN), and 630 mg/kg of total phosphorus (TP). For the DSE-inoculated treatments, the fungal disks (9 mm in diameter, one disk for each pot) were cut from a 14-day-old PDA culture medium and placed 1 cm below the roots of the plants (Ban et al., 2017). For the non-inoculated treatments, the disks (9 mm in diameter) were cut from the PDA culture medium without any fungi in the pot. For the Cd stress treatment, 50 mL of various concentrations of sterilized CdCl_2_ ⋅ 2.5H_2_O solution was added to the culture substrata of each pot to obtain different soil Cd concentrations (5 and 10 mg Cd/kg soil). The control treatments (0 mg Cd/kg soil) were added to 50 mL of sterile water (Wang et al., 2016). All protocols of inoculation and the addition of Cd solution were conducted on a clean bench. All experimental pots were placed in a growth chamber with a 14-h/10-h (day/night) photoperiod at temperatures of 27/22°C and 60% mean air relative humidity. The *M. sativa* and *A. mongolicus* seedlings were harvested at 60 and 90 days after sowing, respectively.

### Plant Biomass and Morphology Traits

At the end of the plant growth promotion experiment, the plant height and the number of leaves were first recorded, and then the shoots and the roots from each pot were harvested separately. The root system was gently washed to remove the adherent sandy soil. The root samples were placed in clear plexiglass trays containing deionized water (approximately 1 cm depth) and scanned by the scanner (EPSON Perfection V800 Photo, Japan). WinRHIZO image analysis system (Regent Instruments, Quebec, QC, Canada) was used to assess the morphological traits, such as the root length, the root surface area, and the root volume. After having been scanned, the roots were collected, and a few root samples were used to analyze the DSE colonization rate (see section “DSE Colonization Analysis”). The fresh shoots and the remaining roots were dried at 70°C for at least 48 h to calculate the plant biomass.

### DSE Colonization Analysis

The cleaned fresh root segments (0.5 cm) were used to detect the DSE colonization according to the method of Phillips and Hayman (1970). The fresh root segments were cleared in 10% (*w*/*v*) potassium hydroxide and then stained in 0.5% (*w*/*v*) acid fuchsin. A total of 50 randomly selected root segments in each sample were observed by microscope at × 20 and × 40 magnification (Biermann and Linderman, 1981). The DSE (total, hyphae, and microsclerotia) colonization rate (%) was expressed as the percentage of the number of infected root segments to the total number of root segments. The DSE colonization intensity was assessed according to Trouvelot et al. (1986) by measuring the percentage of the presence of DSE in all of the intersections observed.

### Determination of Mineral and Cd Mass Fractions in Plant Tissue Samples

The dried plant tissue sample (0.2 g) was digested in a mixed solution including perchloric acid (12.7 mol/L), sulfuric acid (18 mol/L), and water (10:1:2) by the Mars 6 microwave reaction system (CEM Corporation, Matthews, NC, United States) until a transparent solution was obtained. The TN and TP mass fractions of the samples were determined by the Kjeldahl method and vanadium molybdate blue colorimetric method, respectively (Bao, 2000). The organic carbon (OC) mass fraction of the plant tissue sample was determined by the method of dichromate oxidization (Rowell, 1994).

The mixed digestion solution of HNO_3_/HClO_4_ (3:1) (*v*/*v*) was used to digest the dried plant samples (0.2 g) at 200–250°C. The transparent solutions were diluted into a volumetric flask (50 mL) using 0.2% HNO_3_. A flame atomic absorption spectrometer (TAS-990, Beijing Puxi Instrument Factory, Beijing, China) was used to measure the Cd mass fractions in the plant tissues.

### Statistical Analysis

All statistical analyses were performed using SPSS 21.0 software (SPSS Inc., Chicago, IL, United States). The effects of the DSE species, the Cd stress, and their interactions on biomass, SOD activity, soluble protein, GSH, MDA, Cd, and melanin concentration were analyzed by two-way analysis of variance (ANOVA). The effects of the DSE inoculation treatment, the Cd stress treatment, and their interactions on the biomass production, the morphological traits, and the mineral and Cd mass fractions of each plant species were also analyzed by two-way ANOVA. The differences between the means of different treatment groups were analyzed by Duncan’s multiple-range test and were considered to be statistically significant if *P* < 0.05. The values reported in figures are means of at least three replicates. All plots were constructed using the Kaleida Graph 4.5 software (Synergy Software, Reading, PA, United States).

## Results

### Characterization and Identification of DSE Fungi in the Roots of *I. chinensis*

The colonial and microscopic morphology of the two DSEs (DSE1 and DSE2) that were isolated from the roots of *I. chinensis* are illustrated in Supplementary Figure S1. The two DSE colonies were grayish black and black-green, respectively. Although the DSE2 produced chlamydospores (Supplementary Figure S1D), conidial structures were not observed in the DSE1. A comparative analysis of the fungal sequences in the GenBank database identified these DSEs as *A. vagum* and *S. lignicola*. Maximum likelihood analysis clustered the DSE1 (MH410647.1) with *A. vagum* (100% identity with *A. vagum* KY548387.1). Furthermore, the DSE2 (MH410648.1) was grouped in a clade with *S. lignicola* GU586849.1, with a bootstrap support of 100% (Supplementary Figure S2).

### DSE Tolerance to Cd Stress *in vitro*

**FIGURE 1:**
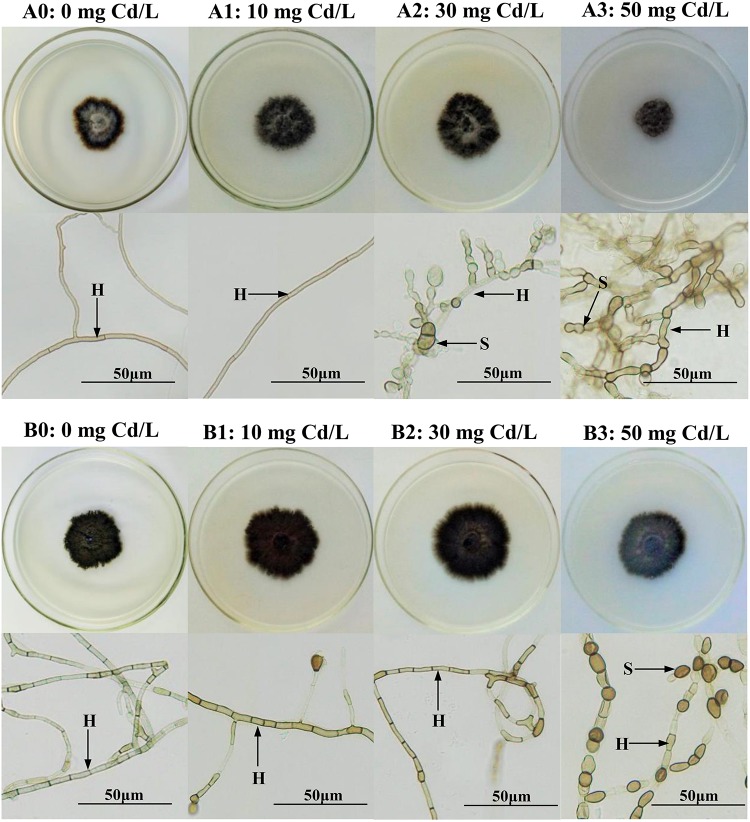
Colonial and microscopic morphology of *Acrocalymma vagum* and *Scytalidium lignicola* in Melin–Norkrans (MMN) medium under varying conditions of Cd stress. **(A0–A3)** Colonies and microscopic morphology of *A. vagum* under varying conditions of Cd stress. **(B0–B3)** Colonies and microscopic morphology of *S. lignicola* under varying levels of Cd stress. *H* DSE hyphae, *S* spore. *Scale bars* = 50 μm.

**FIGURE 2:**
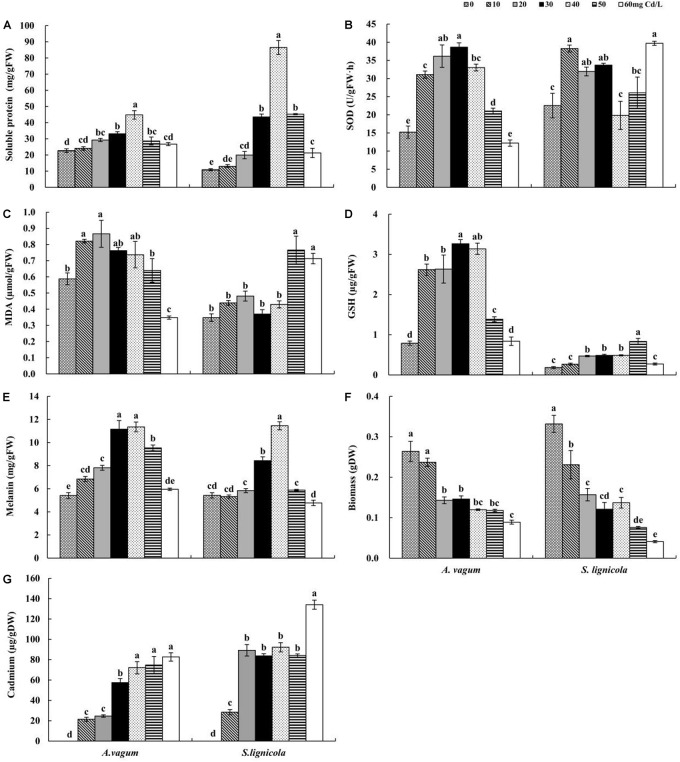
Soluble protein **(A)**, superoxide dismutase (SOD) activity **(B)**, malondialdehyde (MDA) **(C)**, glutathione (GSH) **(D)**, melanin **(E)**, Biomass **(F)**, and cadmium (Cd) **(G)** mass fraction of *Acrocalymma vagum* and *Scytalidium lignicola* under varying levels of Cd stress. The effects of Cd stress were tested by one-way analysis of variance (ANOVA) for each DSE. The *error bars* represent the standard error of the mean (SE). *Different letters* above the error bars indicate a significant difference at *P* < 0.05 by Duncan’s multiple-range test. *FW* indicates fresh weight; *DW* indicates dry weight.

After 14 days of culture, the morphological and microscopic features of the DSE colonies on the culture plates were observed. With enhanced stress treatment, the color of the mycelia appeared to be deeper, and the septal spacing of the mycelia was shorter, while the area of the colony was obviously reduced at a Cd concentration of 50 mg/L. Moreover, the number of chlamydospores was significantly increased at a Cd concentration of 50 mg/L (Figure 1). In liquid media, the biomass and the antioxidant levels of the two DSEs were measured. A two-way ANOVA revealed that the interaction between the DSE and Cd stress had a significant effect on the biomass of the DSE mycelia (Table 1); as the concentration of Cd increased, the biomass decreased (Figure 2F). Generally, the DSEs under evaluation were challenged by Cd stress (Table 1 and Figures 1, 2).

**TABLE 1:** Two-way analysis of variance of the effects of dark septate endophytes (DSEs) and cadmium stress treatment (Cd stress) on biomass, soluble protein, superoxide dismutase (SOD) activity, malondialdehyde (MDA), glutathione (GSH), melanin, and cadmium (Cd) mass fraction of *Acrocalymma vagum* and *Scytalidium lignicola*.

	**Soluble protein (mg/gFW)**		**SOD (U/gFW h)**		**MDA (μmol/gFW)**		**GSH (μg/gFW)**		**Melanin (mg/gFW)**		**Cd (μg/gDW)**		**Biomass (gDW)**	
	***F***	***P***	***F***	***P***	***F***	***P***	***F***	***P***	***F***	***P***	***F***	***P***	***F***	***P***
DSEs	17.8	<*0.001*	9.3	*0.005*	45.9	<*0.001*	686.5	<*0.001*	88.3	<*0.001*	138.4	<*0.001*	0.144	0.707
Cd stress	144.7	<*0.001*	18.3	<*0.001*	5.9	<*0.001*	45.2	<*0.001*	109.6	<*0.001*	165.1	<*0.001*	56.165	<*0.001*
DSEs × Cd stress	49.4	<*0.001*	18.2	<*0.001*	19.5	<*0.001*	38.2	<*0.001*	9.7	<*0.001*	49.5	<*0.001*	3.443	*0.011*

*DSEs and Cd stress represent the two main factors; DSEs indicates the species of DSE (*A. vagum* and *S. lignicola*); Cd stress indicates Cd stress treatment (0, 10, 20, 30, 40, 50, and 60 mg Cd/L); DSEs × Cd stress indicates the interaction effects between DSEs and Cd stress; FW indicates fresh weight; DW indicates dry weight. Significant *P*-values (*P* < 0.05) are in italics.*

As the level of Cd stress increased, the two DSEs showed an increasing trend in the production of soluble protein, GSH, and melanin, which declined after reaching a maximum value at Cd concentrations of 30–50 mg/L (Figures 2A,D,E,G). The highest melanin concentrations of *A. vagum* and *S. lignicola* at 40 mg Cd/L showed increases of 109.6 and 111.0%, respectively, compared with that at 0 mg Cd/L. The soluble protein concentrations of *A. vagum* and *S. lignicola* also showed maximal levels at 40 mg Cd/L, showing significant increases of 97.6 and 694.9%, respectively. The maximum concentrations of GSH in *A. vagum* and *S. lignicola* were observed at 30 and 50 mg Cd/L, respectively, showing significant increases of 313.0 and 353.3%, respectively, compared to that at 0 mg Cd/L. In addition, the Cd mass fractions of both DSEs increased significantly with increasing levels of Cd stress; the maximum level observed was 60 mg Cd/L.

The SOD activity of *A. vagum* showed a trend to increase but then subsequently declined after reaching a maximum of 153.6% at 30 mg Cd/L. For *S. lignicola*, all treatments showed higher levels of SOD activity than that observed at 0 mg Cd/L, except for 40 and 50 mg Cd/L (Figure 2B). The MDA concentration of *A. vagum* increased initially and then decreased with increasing Cd concentration; the concentration of the MDA showed a notable increase at 10 mg Cd/L (+ 39.6%) and 20 mg Cd/L (+ 47.3%) compared with that at 0 mg Cd/L. For *S. lignicola*, the MDA concentration was significantly increased only at concentrations of 50 and 60 mg Cd/L by 118.9 and 103.7% respectively, compared with that at 0 mg Cd/L (Figure 2C).

### DSE Colonization Analysis

**FIGURE 3:**
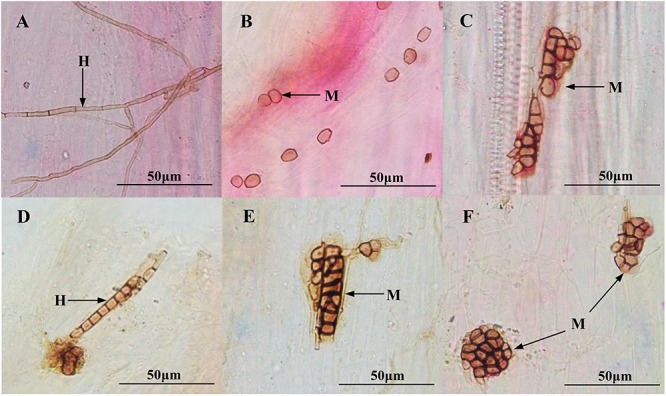
Colonization of dark septate endophytes (DSEs) in the roots of inoculated *Medicago sativa and Ammopiptanthus mongolicus*. **(A)** DSE hyphae in *M. sativa* roots. **(B,C)** DSE microsclerotia in *M. sativa* roots. **(D)** DSE hyphae in *A. mongolicus* roots. **(E,F)** DSE microsclerotia in *A. mongolicus* roots. *H* DSE hyphae, *M* DSE microsclerotia. *Scale bars* = 50 μm.

**FIGURE 4:**
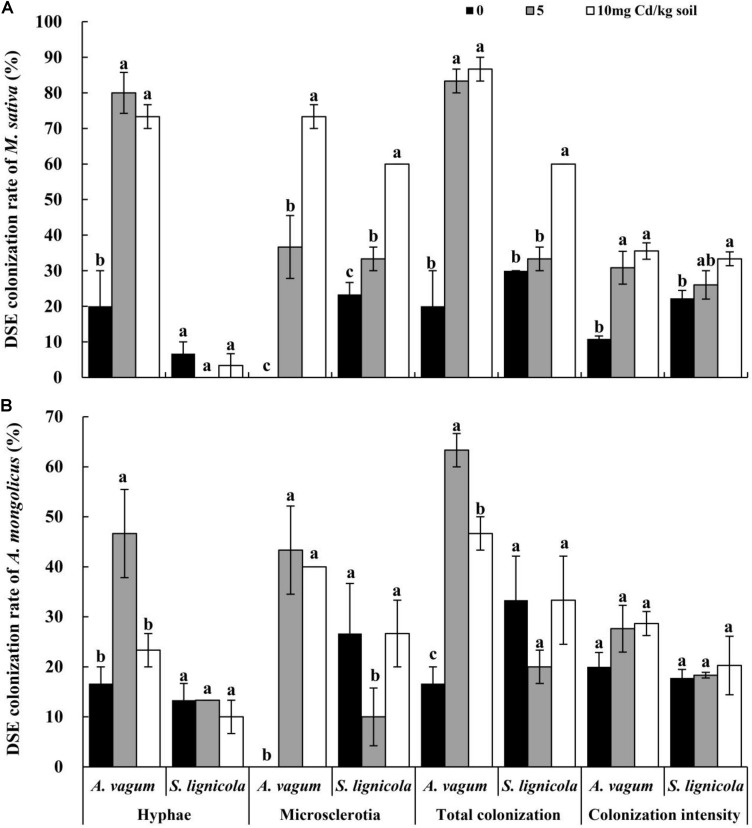
Dark septate endophytes (DSEs) colonization rate in the roots of *Medicago sativa* and *Ammopiptanthus mongolicus* inoculated with DSEs under Cd stress. **(A)** DSEs colonization rate in *M. sati*va roots. **(B)** DSEs colonization rate in *A. mongolicus* roots. The *error bars* represent the standard error of the mean (SE). *Different letters* above the error bars indicate a significant difference at *P* < 0.05 by Duncan’s multiple-range test.

After harvesting, the melanized septate hyphae and the microsclerotia of both DSEs were observed in the inoculated roots of *M. sativa* and *A. mongolica* (Figure 3). For *M. sativa*, the colonization of DSEs was measured at different levels of DSE inoculation and Cd stress treatments (Figure 4A). The colonization of the hyphae ranged from 0 to 80%, the microsclerotial colonization ranged from 0 to 73.3%, the total root colonization ranged from 20 to 86.7%, and the colonization intensity ranged from 10.8 to 35.5%. The inoculation of *A. vagum* significantly increased the DSE colonization in *M. sativa* roots under cadmium stress. Maximal levels of microsclerotial and total colonization and colonization intensity were observed at 10 mg Cd/kg soil; these parameters were all significantly higher than that at 0 mg Cd/kg soil. Maximal levels of hyphal colonization were observed at 5 mg Cd/kg soil and were significantly higher than that at 0 mg Cd/kg soil. The *S. lignicola* inoculation significantly increased the microsclerotial and total colonization and the colonization intensity in *M. sativa* roots at 10 mg Cd/kg soil compared with that at 0 mg Cd/kg soil. However, no significant differences were observed in terms of hyphal colonization.

For *A. mongolicus* (Figure 4B), the hyphal colonization ranged from 10 to 46.7%, the microsclerotial colonization ranged from 0 to 43.3%, the total colonization ranged from 16.7 to 63.3%, and the colonization intensity ranged from 17.8 to 28.7%. The *A. vagum* inoculation increased the DSE colonization in the *A. mongolicus* roots under Cd stress. Maximal values of hyphal, microsclerotial, and total colonization were significantly higher at 5 mg Cd/kg soil than at 0 mg Cd/kg soil. The *S. lignicola* inoculation significantly decreased the microsclerotial colonization at 5 mg Cd/kg soil compared with other Cd concentrations. No significant differences were found in terms of the hyphal or total colonization and the colonization intensity when compared across the different Cd concentrations.

### Plant Morphology and Development

**FIGURE 5:**
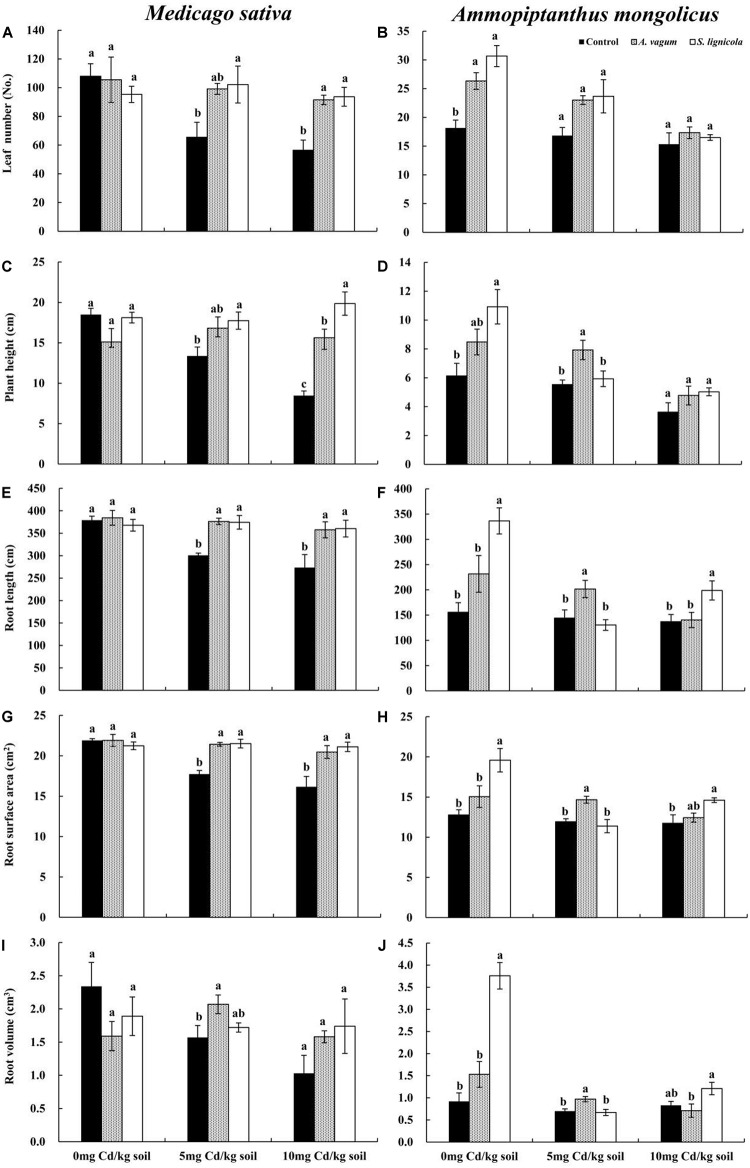
Leaf number of *Medicago sativa* **(A)**, Leaf number of *Ammopiptanthus mongolicus* **(B)**, Plant height of *M. sativa* **(C)**, Plant height of *A. mongolicus* **(D)**, Root length of *M. sativa* **(E)**, Root length of *A. mongolicus* **(F)**, Root surface area of *M. sativa* **(G)**, Root surface area of *A. mongolicus* **(H)**, Root volume of *M. sativa* **(I)**, and Root volume of *A. mongolicus* **(J)**. The effects of dark septate endophytes (DSEs) inoculation and Cd stress treatment on the morphology of *Medicago sativa* and *Ammopiptanthus mongolicus*. Parameters were analyzed separately for each plant species. The *error bars* represent the standard error of the mean (SE). *Different letters* above the error bars indicate a significant difference at *P* < 0.05 by Duncan’s multiple-range test. *Control* indicates non-inoculated plants. *A. vagum* indicates the plants inoculated with *Acrocalymma vagum*. *S. lignicola* indicates the plants inoculated with *Scytalidium lignicola*.

The plant height, the root length, and the root surface area of *M. sativa* were significantly affected by the DSE inoculation, the Cd stress, and their interactions (Table 2). The *A. vagum* and *S. lignicola* inoculation reduced the negative effects of increased Cd stress on plants (Figures 5A,C,E,G,I). At 0 mg Cd/kg soil, there were no significant differences in the morphology of *M. sativa* when compared between the inoculated (*A. vagum* and *S. lignicola*) and the non-inoculated treatments. At 5 mg Cd/kg soil, the root morphology of *M. sativa* plants was significantly and positively affected by the inoculation with *A. vagum* compared to the non-inoculated treatment. In the inoculated *S. lignicola* plants, the significantly positive effects were observed on the leaf number, the plant height, the root length, and the root surface area. At 10 mg Cd/kg soil, there was a significant increase in the morphology of *M. sativa* plants inoculated with *A. vagum* and *S. lignicola* when compared to the non-inoculated treatment.

**TABLE 2:** Two-way analysis of variance of the effects of dark septate endophytes (DSEs) inoculation and cadmium stress treatment (Cd stress) on plant morphology and biomass production of *Medicago sativa* and *Ammopiptanthus mongolicus.*

	**Leaf number**		**Plant height (cm)**		**Shoot biomass (gDW)**		**Root biomass (gDW)**		**Root/shoot ratio**		**Root length (cm)**		**Root surface area (cm^2^)**		**Root volume (cm^3^)**	
	***F***	***P***	***F***	***P***	***F***	***P***	***F***	***P***	***F***	***P***	***F***	***P***	***F***	***P***	***F***	***P***
***M. sativa***																
DSEs	5.4	*0.014*	15.3	<*0.001*	7.2	*0.005*	0.8	0.461	3.3	0.061	10.7	*0.001*	16.1	<*0.001*	0.3	0.774
Cd stress	4.6	*0.024*	3.9	*0.04*	7.7	*0.004*	10.9	*0.001*	2.0	0.165	6.3	*0.008*	10.2	*0.001*	2.9	0.079
DSEs × Cd stress	2.7	0.066	8.5	<*0.001*	8.8	<*0.001*	1.4	0.26	2.9	0.051	3.1	*0.042*	5.5	*0.004*	2.6	0.071
***A. mongolicus***																
DSEs	15.0	<*0.001*	8.4	*0.003*	2.6	0.099	2.7	0.092	1.3	0.309	10.5	*0.001*	9.3	*0.002*	30.4	<*0.001*
Cdstress	21.7	<*0.001*	23.4	<*0.001*	14.8	<*0.001*	11.1	*0.001*	5.9	*0.011*	16.1	<*0.001*	12.0	<*0.001*	49.7	<*0.001*
DSEs × Cd stress	3.1	*0.04*	3.4	*0.031*	2.3	0.095	4.8	*0.008*	6.2	*0.002*	7.6	*0.001*	6.7	*0.002*	23.2	<*0.001*

*DSEs and Cd stress represent the two main factors; DSEs indicates DSE inoculation treatment (non-inoculated control, *A. vagum* and *S. lignicola*); Cd stress indicates Cd stress treatment (0, 5, and 10 mg Cd/kg soil); DSEs × Cd stress indicates the interaction effects between DSEs and Cd stress; DW indicates dry weight. Significant *P*-values (*P* < 0.05) are in italics.*

The morphology of *A. mongolicus* plants was significantly affected by the DSE inoculation, the Cd stress, and their interactions (Table 2). Although inoculation with *A. vagum* or *S. lignicola* was beneficial to *A. mongolicus*, the positive effects were reduced with increasing concentrations of Cd (Figures 5B,D,F,H,J). At 0 mg Cd/kg soil, the *A. vagum* inoculation caused a significant increase in leaf number compared to that observed in the non-inoculated treatment. The *S. lignicola* inoculation significantly increased the morphology of *A. mongolicus* plants compared to the non-inoculated treatment. At 5 mg Cd/kg soil, the *A. vagum* inoculation significantly improved the plant height, the root length, the root volume, and the root surface area of *A. mongolicus* plants compared to the non-inoculated treatment; however, no significant differences were found in the plants subjected to *S. lignicola* inoculation. At 10 mg Cd/kg soil, the *S. lignicola* inoculation significantly increased the root surface area and the root length by 24.4 and 45.1%, respectively, compared with the non-inoculated treatment.

### Plant Biomass Production

**FIGURE 6:**
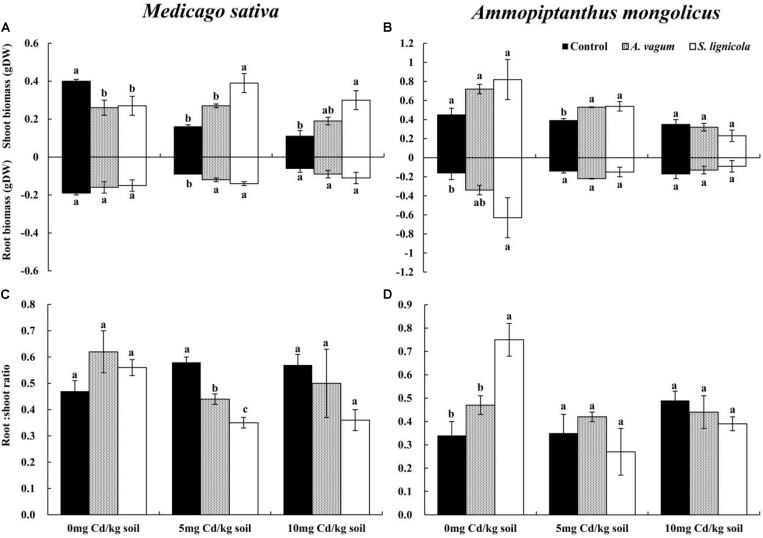
Shoot biomass and root biomass of *Medicago sativa* **(A)**, shoot biomass and root biomass of *Ammopiptanthus mongolicus* **(B)**, Root/shoot ratio of *M. sativa* **(C)**, and Root/shoot ratio of *A. mongolicus* **(D)**. The effects of dark septate endophytes (DSEs) inoculation and Cd stress treatment on the biomass production and root/shoot ratio of *Medicago sativa* and *Ammopiptanthus mongolicus*. Parameters were analyzed separately for each plant species. The *error bars* represent the standard error of the mean (SE). *Different letters* above the error bars indicate a significant difference at *P* < 0.05 by Duncan’s multiple-range test. *Control* indicates non-inoculated plants. *A. vagum* indicates the plants inoculated with *Acrocalymma vagum*. *S. lignicola* indicates the plants inoculated with *Scytalidium lignicola*. *DW* indicates dry weight.

A two-way ANOVA indicated significant interaction effects of the DSEs by Cd stress on the shoot biomass of *M. sativa*. Furthermore, Cd stress had a significant effect on root biomass, while no interaction effects were observed on the root/shoot ratio (Table 2). Cd stress clearly inhibited the biomass of *M. sativa*; however, this pressure was alleviated by the inoculation with the two DSE fungi. At 5 mg Cd/kg soil, the *A. vagum* inoculation significantly improved the root biomass (+ 33.3%) of *M. sativa*, while *S. lignicola* promoted both shoot (+ 143.8%) and root biomass (+ 55.6%). Generally, both the *A. vagum* and *S. lignicola* inoculation significantly affected the root/shoot ratio. At 10 mg Cd/kg soil, the *A. vagum* inoculation had a positive influence on the shoot and root biomass but a negative effect on the root/shoot ratio. The *S. lignicola* inoculation significantly promoted the shoot biomass (+ 172.7%) of *M. sativa*; however, no significant differences were found in terms of the root biomass and the root/shoot ratio (Figures 6A,C).

For *A. mongolicus*, significant interaction effects of the DSE and Cd stress were observed on the root biomass and the root/shoot ratio; the shoot biomass was significantly affected by Cd stress (Table 2). At 0 mg Cd/kg soil, the *S. lignicola* inoculation significantly improved the root biomass (+ 293.8%) and the root/shoot ratio (+ 120.6%) of *A. mongolicus* plants compared to the non-inoculated treatment. At 5 mg Cd/kg soil, the *A. vagum* and *S. lignicola* inoculation significantly improved the shoot biomass (35.9 and 38.5%) of plants compared with those that were not inoculated. At 10 mg Cd/kg soil, there were no significant differences in terms of the plant biomass and the root/shoot ratio among the plants inoculated with *A. vagum* and *S. lignicola* when compared with the non-inoculated treatment (Figures 6B,D).

### Mineral and Cd Mass Fractions in Plant Tissue Samples

**FIGURE 7:**
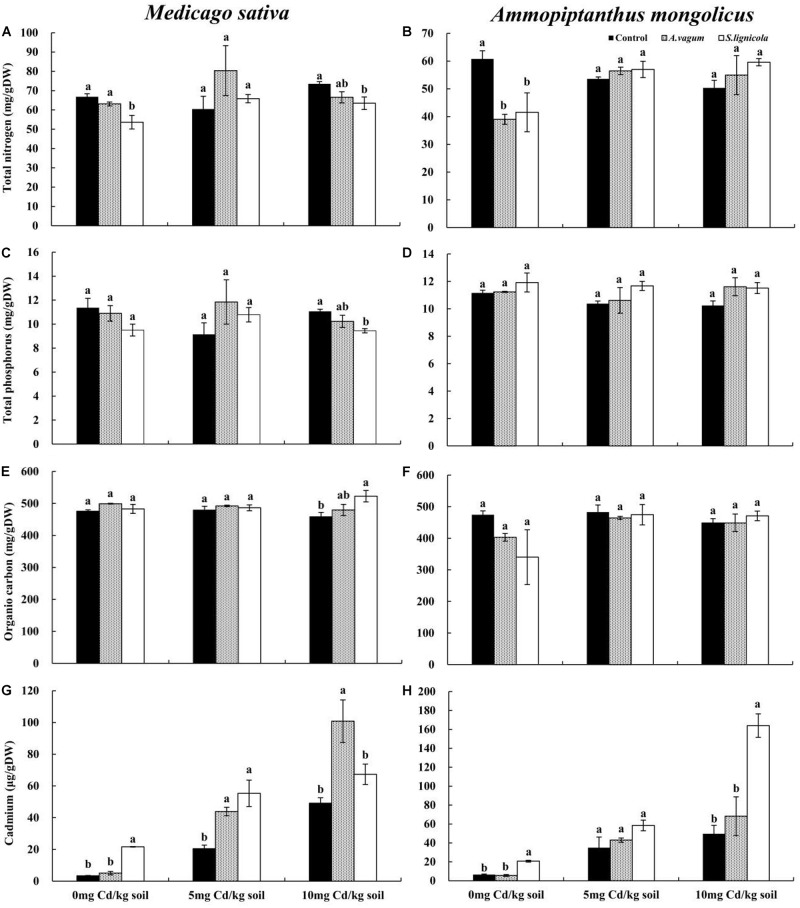
Total nitrogen of *Medicago sativa* **(A)**, total nitrogen of *Ammopiptanthus mongolicus* **(B)**, total phosphorus of *M. sativa* **(C)**, total phosphorus of *A. mongolicus* **(D)**, organic carbon of *M. sativa* **(E)**, organic carbon of *A. mongolicus* **(F)**, cadmium of *M. sativa* **(G)**, and cadmium of *A. mongolicus* **(H)**. The effects of dark septate endophytes (DSEs) inoculation and Cd stress treatment on the mineral and Cd mass fractions of *M. sativa* and *A. mongolicus*. Parameters were analyzed separately for each plant species. The *error bars* represent the standard error of the mean (SE). *Different letters* above the error bars indicate a significant difference at *P* < 0.05 by Duncan’s multiple-range test. Control indicates non-inoculated plants. *A. vagum* indicates the plants inoculated with *Acrocalymma vagum*. *S. lignicola* indicates the plants inoculated with *Scytalidium lignicola*. *DW* indicates dry weight.

A two-way ANOVA (Table 3) revealed significant interaction effects of the DSE inoculation by Cd stress on the Cd mass fractions of *M. sativa*. Under Cd stress, the *A. vagum* and *S. lignicola* inoculation had a positive and significant effect on Cd accumulation in the *M. sativa* plants when compared to the non-inoculated treatment (Figure 7G). Maximum values of Cd mass fractions in the *M. sativa* plants inoculated with *A. vagum* or *S. lignicola* were observed at 10 mg Cd/kg soil. The increase was significant for *A. vagum* and resulted in 15.15 μg Cd per colonized plant compared to 4.55 μg Cd per control plant. Furthermore, Cd stress significantly affected the OC mass fraction of *M. sativa*. The *S. lignicola* inoculation significantly increased the OC (+ 14%) of *M. sativa* plants at 10 mg Cd/kg soil compared to the non-inoculated treatment (Figure 7E). *A. vagum* and *S. lignicola* inoculation had a positive effect on TN and TP in *M. sativa* plants at 5 mg Cd/kg soil (Figures 7A,C).

**TABLE 3:** Two-way analysis of variance for the effects of dark septate endophytes (DSEs) inoculation and cadmium stress treatment (Cd stress) on total nitrogen (TN), total phosphorus (TP), organic carbon (OC), and cadmium (Cd) mass fractions of *Medicago sativa* and *Ammopiptanthus mongolicus*.

	**TN (mg/gDW)**		**TP (mg/gDW)**		**OC (mg/gDW)**		**Cd (μg/gDW)**	
	***F***	***P***	***F***	***P***	***F***	***P***	***F***	***P***
***M. sativa***								
DSEs	2.3	0.134	1.3	0.307	4.0	*0.037*	17.4	<*0.001*
Cd stress	1.9	0.183	0.2	0.847	0.0	0.993	83.2	<*0.001*
DSEs × Cd stress	2.1	0.127	1.8	0.165	2.5	0.076	7.0	*0.001*
***A. mongolicus***								
DSEs	1.2	0.328	3.7	*0.045*	1.1	0.353	24.3	<*0.001*
Cd stress	4.7	*0.023*	0.9	0.422	3.2	0.065	57.3	<*0.001*
DSEs × Cd stress	5.2	*0.006*	0.6	0.655	1.5	0.252	9.7	<*0.001*

*DSEs and Cd stress represent the two main factors; DSEs indicates DSE inoculation treatment (non-inoculated control, *A. vagum* and *S. lignicola*); Cd stress indicates Cd stress treatment (0, 5, and 10 mg Cd/kg soil); DSEs × Cd stress indicates the interaction effects between DSEs and Cd stress; DW indicates dry weight. Significant *P*-values (*P* < 0.05) are in italics.*

A two-way ANOVA (Table 3) showed significant interaction effects of the DSE inoculation and Cd stress on the Cd and TN mass fractions of *A. mongolicus*. Under maximal Cd stress, the inoculation with *S. lignicola* significantly increased the Cd mass fractions compared to the control plants (Figure 7H). This resulted in 51.12 μg Cd per colonized plant compared to 30.60 μg Cd per control plant. In addition, the *A. vagum* and *S. lignicola* inoculation had a positive effect on the TN and TP mass fractions of *A. mongolicus* under Cd stress. The *A. vagum* inoculation increased the TN mass fractions (44.8 and 40.9%) of *A. mongolicus* in the 5 and 10 mg Cd/kg soil treatments compared with those subjected to the 0 mg Cd/kg soil treatments. Moreover, the *S. lignicola* inoculation also increased the TN mass fractions (+ 37.2 and + 43.5%) in the 5 and 10 mg Cd/kg soil treatments, respectively, compared with those subjected to the 0 mg Cd/kg soil treatment (Figure 7B). *A. vagum* and *S. lignicola* inoculation had a positive effect on TP and OC in *A. mongolicus* plants at 10 mg Cd/kg soil (Figures 7D,F).

## Discussion

### DSE Tolerance to Cd Stress

The DSE fungi, *A. vagum* and *S. lignicola*, are widely distributed in ecosystems, such as forests and crops, and their positive effects on host plants have been widely reported (Zhao et al., 2014; Jin et al., 2018; Maulana et al., 2018). In this study, *A. vagum* and *S. lignicola* were first isolated from *I. chinensis* in areas contaminated with heavy metals to determine the Cd tolerance of the DSE fungi and the response of the non-host plants to DSE inoculation. Previous studies have reported that strains with a high tolerance to heavy metals are more likely to be isolated from regions that are contaminated by heavy metals (Xu et al., 2015). The present study indicated that the biomass of *A. vagum* and *S. lignicola* declined with increasing Cd stress and showed that a concentration of 10 mg Cd/L was more beneficial for DSE growth. We speculate that the performance of the DSEs under Cd stress might be correlated with their original Cd-polluted environments (5.4–11.1 mg Cd/kg soil) and the ability of *I. chinensis* to tolerate Cd stress in the Fengfeng mining area (Yu et al., 2019). However, we observed a trend for a significant increase in the Cd mass fraction of both DSEs with increasing levels of Cd stress; these findings are consistent with those reported previously by Duddridge and Wainwright (1980). Previous studies have also shown that the DSEs can chelate or bind heavy metal ions using cell wall constituents in the extracellular tissues. In addition, heavy metal ions are complexed, bound to peptides, and compartmentalized in the intracellular tissues by the DSEs in order to reduce the adverse effects of heavy metals (Zhao et al., 2015; Shakya et al., 2016).

Generally, heavy metal stress usually exerts negative effects on organisms and causes oxidative damage in the cells (Gallego et al., 2012; Nannoni et al., 2016). In the present study, the SOD activity, MDA, soluble protein, GSH, and melanin concentrations in the DSE fungi were evaluated to determine the effects of Cd stress on the antioxidant substances. SOD is considered to be an important enzyme that can eliminate the reactive oxygen species (ROS) that are generated under conditions of oxidative damage (Collin-Hansen et al., 2005). We found that the activity of SOD in the DSEs was elevated under Cd stress (*A. vagum*: 10–50 mg Cd/L, *S. lignicola*: 10–30, and 50–60 mg Cd/L). These results showed that, under high levels of Cd stress, SOD began to accumulate in order to scavenge ROS and therefore minimize oxidative damage. Previous studies also reported increased levels of SOD activity in the DSEs under Pb and Cd stress (Ban et al., 2012; Zhan et al., 2015a). In addition, enhancement in the SOD activity of the DSEs has also been reported in relation to salinity stress (Pan et al., 2018). Under stressful conditions, MDA acts as a biomarker for oxidative stress and can be used to assess the extent of damage caused by oxidative stress (Khan et al., 2012). The accumulation of MDA in *A. vagum* at a concentration of 10–20 mg Cd/L and *S. lignicola* at a concentration of 50–60 mg Cd/L clearly demonstrated that the DSE strains were adversely affected by Cd stress. Soluble protein, as a compatible osmolyte, can facilitate osmotic adjustment and help to increase tolerance to dehydration (Doganlar et al., 2010). The accumulation of soluble protein in *A. vagum* and *S. lignicola* reduced the negative influences of Cd stress by counterbalancing the solute potential, thus contributing to cell growth. By virtue of its ability to alleviate oxidative damage, melanin has been shown to enhance the survival of the DSEs in stressful habitats with harsh conditions (Eisenman and Casadevall, 2012; Fernandez and Koide, 2013). Therefore, as observed in the current study, an increase in the melanin concentration in the 10–50 mg Cd/L treatment with *A. vagum* and the 30–40 mg Cd/L treatment with *S. lignicola* may have contributed to their resistance to Cd stress.

In the present study, the concentrations of GSH in *A. vagum* treated with 10–50 mg Cd/L and *S. lignicola* treated with 20–50 mg Cd/L were markedly increased compared to the control treatments. Among thiol compounds, GSH is a tripeptide with a -SH group and acts as a key compound in mitigating Cd-induced damage by playing a fundamental dual role as an antioxidant and a ligand peptide (Sobrino-Plata et al., 2014; Hernández et al., 2015).

### Effects of DSE Inoculation on Non-host Plants

Research has shown that endophytic fungi inhabit most plant roots in habitats polluted by Cd (Li et al., 2012; Yu et al., 2019). The significant effects of the endophytic fungi on plant growth and Cd tolerance have also been demonstrated (He et al., 2017; Zhan et al., 2017). In the present study, the hyphae and the microsclerotial structures of the DSEs were observed in all inoculated *M. sativa* and *A. mongolicus* roots after harvesting. Our results showed that both DSEs under evaluation can be effective root colonizers, even under Cd stress. Moreover, these results confirmed that the DSEs have broad host specificity, spanning herbs to woody species (Berthelot et al., 2016). Under high concentrations of Cd, we found that the colors of the colonies and the hyphae were deeper, the septal spacing of the mycelia was shorter, and the colony area was also reduced. In addition, microscopy revealed that the two DSE strains under investigation could infect and form dark septate hyphae and microsclerotia in the intracellular and intercellular tissues of *M. sativa* and *A. mongolicus* roots. Furthermore, we also identified that these DSEs exhibited colonization predominantly by melanized hyphae. These hyphae not only grow inter- and intra-cellularly within the cortex but also extend into the vascular tissue through their ability to traverse plant cell walls, which provide a beneficial means of exchanging nutrients between the hyphae and the plants (Addy et al., 2005; Peterson et al., 2008; Li et al., 2015). Furthermore, we also found that the microsclerotia showed high levels of colonization. In a previous study, Peterson et al. (2008) reported that the microsclerotia should be considered as propagules or hypopus. The chlamydospore-like structures that were observed *in vitro* may also be an important strategy that can be utilized by the DSE fungi to resist Cd stress in environments polluted by heavy metals. Moreover, the response of *M. sativa* and *A. mongolicus* to the DSE colonization was strain dependent but was independent on the concentration of Cd. Specifically, the inoculation of *M. sativa* with *S. lignicola* led to a significantly higher shoot biomass than that of the controls at concentrations of 5 and 10 mg Cd/kg soil. Furthermore, the inoculation of *M. sativa* with *A. vagum* showed a significantly higher root biomass than that of the controls at a concentration of 5 mg Cd/kg soil, whereas the *A. mongolicus* plants inoculated with either of the two DSE strains showed a significantly higher shoot biomass at 5 mg Cd/kg soil. Previous studies (Ban et al., 2012; Berthelot et al., 2016) have shown that the influence of the DSE inoculation on plant growth varies under Cd stress and that the DSE–plant interactions depend on the DSE species involved. These previous findings are consistent with our present results.

The inoculation with DSEs was also shown to regulate the root architecture of both *M. sativa* and *A. mongolicus* to improve plant performance and tolerance to Cd. In the present study, significantly higher root length and surface area were observed in the two plants inoculated with *A. vagum* and *S. lignicola* under Cd stress when compared with the control plants. These findings indicate that the inoculation of the DSEs can facilitate the growth of root systems. The development of a deep and extensive root system can promote water absorption and nutrient uptake by plants, which eventually affects the production of biomass (Li et al., 2018). Wu et al. (2010) evaluated the effects of the DSE inoculation in an endangered Chinese medicinal plant and found that the DSEs could stimulate root development under non-stressful situations. Li et al. (2018) also reported that the DSE inoculation enhanced the branch growth and root biomass of *A. mongolicus*, which could facilitate the adaptation of this plant to arid environments. Thus, the larger root surface area and the longer root length of *M. sativa* and *A. mongolicus* observed in the present study are probably beneficial for the adaptation of these plants to environments polluted by heavy metals (Mayerhofer et al., 2013).

Previous studies have indicated that the DSE inoculation can increase the biomass and the mineral levels of mineral nutrition (Newsham, 2011; Li et al., 2018). In the present study, for *M. sativa*, only inoculation with *S. lignicola* led to an increase in the OC mass fraction, whereas *A. mongolicus* plants inoculated with either *A. vagum* or *S. lignicola* led to positive effects on the TN levels under Cd stress. Our findings indicate that the DSEs may have the potential to increase the absorption of nitrogen under Cd stress. The beneficial effects of endophytic fungi on nitrogen absorption and nitrogen fixation in plants have been demonstrated previously (Newsham, 2011; Wei et al., 2016; Liu et al., 2019). Previous studies have demonstrated that the AM fungi can promote nitrogen uptake in plant roots and revealed the substantially positive effects on nodulation and nitrogen fixation in legumes (Singh, 1996; Antunes et al., 2006; Yang et al., 2016). The DSEs are considered to exert ecological functions that are similar to those of the AM fungi and may play essential roles in nitrogen absorption, nodulation, and nitrogen fixation in leguminous plants, particularly in stressful environments. Further research on the effects of the DSEs on nodulation and nitrogen fixation in non-host leguminous plants will facilitate a better understanding of the ecological function of the DSEs in stressful ecosystems, such as those contaminated by heavy metals. Interestingly, the inoculation of the two DSE strains selected for investigation herein led to an enhancement in the Cd accumulation of *M. sativa* and *A. mongolicus* plants. Specifically, inoculation with *A. vagum* significantly increased the Cd accumulation in *M. sativa* by 232.97% per plant under 10 mg Cd/kg soil, and the *S. lignicola* inoculation had a significant effect on the Cd accumulation in *A. mongolicus* (67.06% increase per plant). Our findings therefore indicate that the DSEs can promote the growth and the tolerance of plants to Cd stress. Moreover, the accumulation of Cd in the mycelia of the DSEs may represent an important strategy by which plants may be able to resist Cd stress. This is particularly crucial for bio-remediation in areas that are polluted by heavy metals. Similar phenomena have been reported in previous studies (Berthelot et al., 2016; He et al., 2017). A previous study showed that the endophyte *Sphingomonas* SaMR12 could increase the concentration of GSH by upregulating the expression of relevant genes and subsequently increasing Cd accumulation and tolerance in the host plant (Pan et al., 2016). Zhu et al. (2018) reported that the DSEs can increase the activity of antioxidant enzymes and reduce the levels of heavy metals in roots and shoots, thereby improving the growth and the tolerance of tomato plants under heavy metal stress. In addition, the positive effects of the DSEs on the growth of maize crops were previously shown to be affected by the photosynthetic activity, the regulation of phytohormone balance, and the reduction of Cd levels in the leaves (He et al., 2017). Therefore, it is clear that the complex mechanism by which the DSEs promote the growth and the tolerance of plants to heavy metal stress still requires further research.

## Conclusion

In the present study, we discovered that two DSEs isolated from *I. chinensis* are effective root colonizers of a non-host herb (*M. sativa*) and woody plant (*A. mongolicus*). Although these DSEs did not exhibit high levels of Cd tolerance, they did exhibit certain growth capability under Cd stress *in vitro*. The DSE inoculation significantly improved the growth and the Cd accumulation of non-host plants in soils that were undergoing Cd stress. However, this favorable effect was dependent on host affiliation, DSE species, and Cd concentration. In particular, the two plants being colonized accumulated significantly more Cd than the corresponding controls under Cd stress, which improves the remediation activity of these plants. Thus, both DSEs could be used to promote the cultivation of forage grass and ornamental plants and facilitate the remediation of contaminated soils and ecological restoration. Further investigation is still required on the resources and the functions of different DSE species and their association with different plant species to determine their specific ecological roles under heavy metal stress treatment.

## Data Availability Statement

All datasets generated for this study are included in the article/Supplementary Material.

## Author Contributions

JY, LH, LZ, and XH conceived and designed the experiments. JY and LH performed the experiments and analyzed the data. XH and LH wrote the manuscript.

## Conflict of Interest

The authors declare that the research was conducted in the absence of any commercial or financial relationships that could be construed as a potential conflict of interest.
