# Supplementary material for: Dark Septate Endophytes Improve the Growth and the Tolerance of Medicago sativa and Ammopiptanthus mongolicus Under Cadmium Stress
Source: Front Microbiol. 2020 Jan 28;10:3061. doi: 10.3389/fmicb.2019.03061 (PMC6997539; doi:10.3389/fmicb.2019.03061)
Supplement: Supplementary file 1 [file Data_Sheet_1.pdf]

Supplementary Material

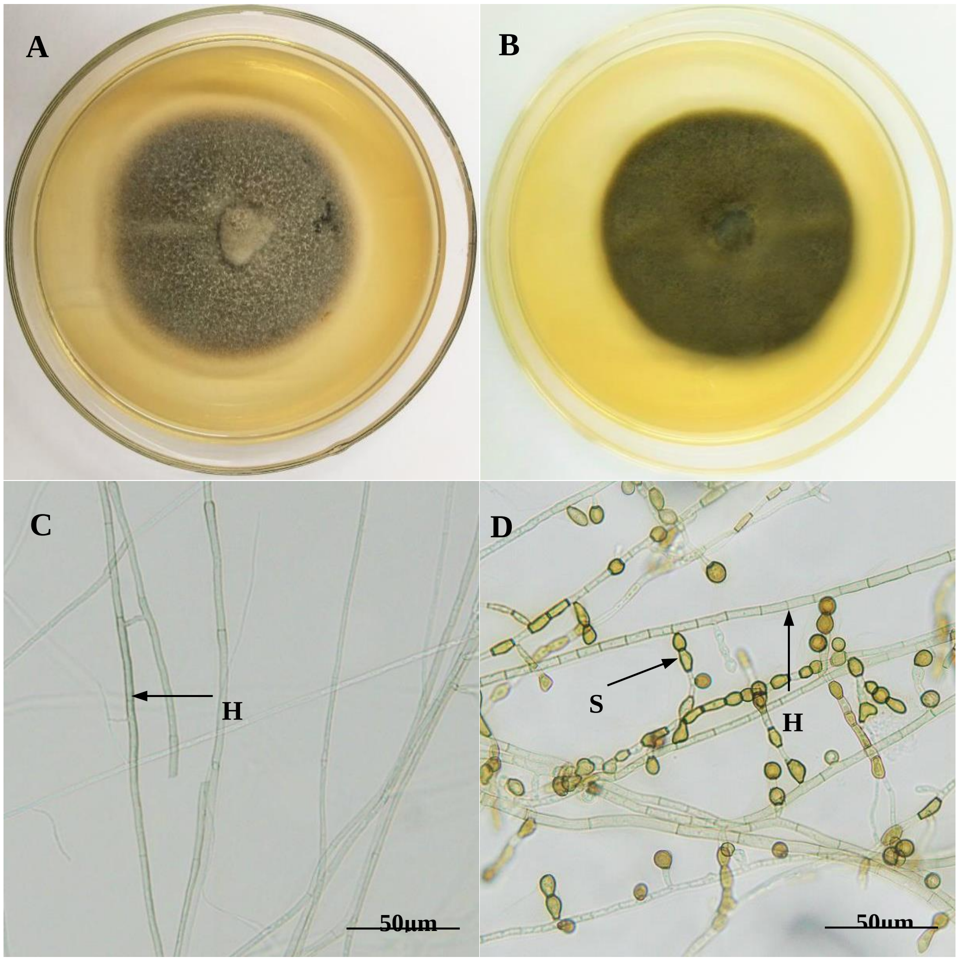

Figure S1 Colonies of endophytic fungi isolated from the roots of *Ilex chinensis* (**A**, **B**). Microscopic morphology of endophytic fungi (**C**, **D**) (Scale bars =50 μm). **A**, **C**: *Acrocalymma vagum*; **B**, **D**: *Scytalidium lignicola*. Arrows indicate: H=DSE hyphae, S=DSE spore.

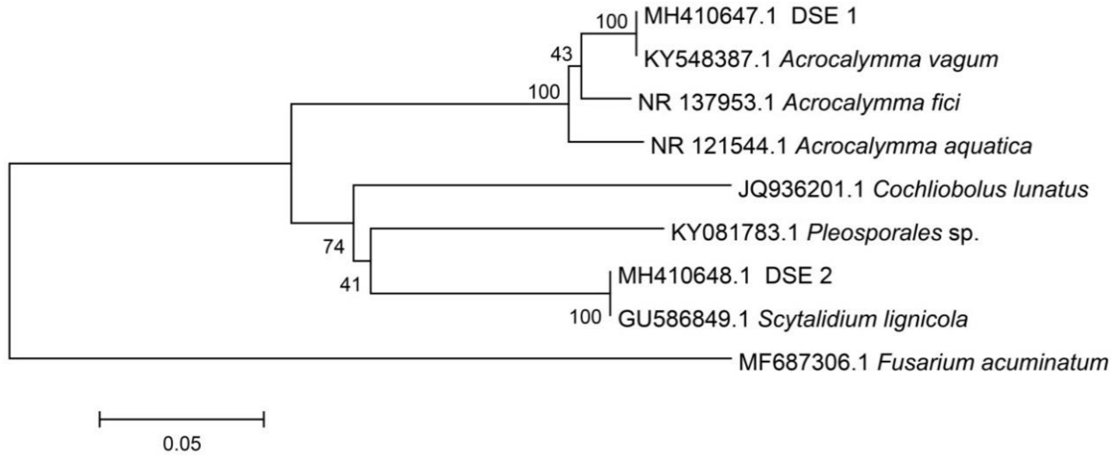

Figure S2 Maximum Likelihood tree based on nrDNA ITS region sequences of DSE isolated from *Ilex chinensis*. Numbers (%) on the main branches represent bootstrap support values (for 1000 iterations). The scale bar shows a distance equal to 5% nucleotide diversity.
